# Quantifying the Motivational Effects of Cognitive Fatigue Through Effort-Based Decision Making

**DOI:** 10.3389/fpsyg.2018.00843

**Published:** 2018-05-30

**Authors:** Stijn A. A. Massar, Árpád Csathó, Dimitri Van der Linden

**Affiliations:** ^1^Centre for Cognitive Neuroscience, Duke-NUS Medical School, Singapore, Singapore; ^2^Institue of Behavioral Sciences, Medical School, University of Pécs, Pécs, Hungary; ^3^Department of Psychology, Education, and Child Studies, Erasmus University, Rotterdam, Netherlands

**Keywords:** fatigue, motivation, performance, cognitive effort, effort-based decision-making

Prolonged active engagement on cognitively demanding tasks often leads to a subjective state labeled cognitive fatigue (Meijman, 1991; Lorist et al., 2000). Although such fatigue is considered to be a complex, multifaceted state involving various causes and effects, it is widely acknowledged that reduced motivation for effort is one of its key aspects (van der Linden, 2011). Accordingly, there seems to be agreement that performance deficits in fatigue are likely to reflect a combination of reduced capacity and reduced willingness to perform (Kanfer and Ackerman, 1989; Meijman, 1991; Hockey, 1997). Despite such consensus, however, only a handful of studies have explicitly targeted the motivational factors that determine performance levels during fatigue. A potential reason for this lack of formal studies could be that motivation is particularly difficult to measure in other ways than by self-report, with the obvious drawbacks that subjects may not always be willing to report loss of motivation or may not be aware of it. In this paper, we argue that recently developed methods and insights from the field of effort-based decision making may help to elucidate how fatigue changes the motivation to perform (Chong et al., 2016; Pessiglione et al., 2017). We will discuss the parallels between theoretical models of fatigued performance and models of effort-based decision making. Further, we will discuss how methods from the effort-based decision-making field can be used to study motivational decline in fatigue-related conditions.

## Fatigue, performance and effort: an old tradition

Researchers as early as Thorndike (1900) have observed that performance decline due to fatigue may depend on a reduced desire to exert further effort. A wide range of earlier theoretical models have proposed that performance critically depends on the motivated allocation of processing resources (Bartley and Chute, 1947; Kahneman, 1973; Kanfer and Ackerman, 1989; Hockey, 1997). Under fatigue, the total available resources may decline, even though that is still a matter of debate (Inzlicht et al., 2014; Christie and Schrater, 2015). More relevant here, however, is that fatigue may also act to shift performance priorities. The Motivational Control Model by Hockey (1997, 2011) describes how performance under demanding conditions (e.g., stress, fatigue) depends on mobilizing required cognitive resources. If task goals are deemed sufficiently important, allocation of such resources can be channeled through exertion of compensatory effort. Yet, this comes at the expense of increased discomfort. Alternatively, task goals could be adjusted or even abandoned. Management of effort allocation and goal selection would be arbitrated by higher-order control functions that take input from effort and goal monitoring mechanisms. A model by Boksem and Tops (2008) takes a biological perspective, and describes how effort allocation relies on a constant monitoring of the energetic costs of performance, weighted against the value of its outcomes (e.g., food or monetary reward obtained). Several brain areas are proposed to coordinate effort monitoring (e.g., anterior insula), reward (e.g., nucleus accumbens) and action outcomes (e.g., anterior cingulate cortex). Actions are only engaged when task goals are deemed sufficiently important. Both these models imply some degree of volitional regulation of resource allocation based on internal cost-benefit weighing mechanism.

This idea has been further formalized in the Integrated Resource-Allocation model by Kanfer and Ackerman (1989) and Kanfer (1990, 2011), stating that the relationships between effort, performance, and outcome value can be expressed in subjective utility functions. These functions would describe how the subjective utility of outcomes increases with better performance, and decreases with increased effort exertion. Decisions on how much effort to exert would depend on finding the optimal balance between effort, performance and utility. Under fatigue, the disutility of effort would increase, leading to less allocation of effort (Kanfer, 2011). Two more recent models similarly describe how performance levels relate to weighted decisions based on the value of the task at hand, versus the value of alternative action options (opportunity cost; Kurzban et al., 2013) (self-control depletion; Inzlicht et al., 2014).

Given the general emphasis on effort allocation in fatigue, it is surprising that only very few empirical studies have targeted this area directly. Some studies showed that motivational incentives (e.g., monetary reward) can lead to improved performance under fatigue (Boksem et al., 2006; Hopstaken et al., 2014, 2015, 2016; but see Gergelyfi et al., 2015). A different approach was applied by Holding and colleagues (Shingledecker and Holding, 1974; Holding et al., 1983) who assessed effort allocation through a decision-making paradigm. To complete a task (detecting a fault in an electrical circuit), participants could choose between an effortful strategy (checking multiple circuits) with a higher probability of correct performance, or a less effortful strategy (checking only one circuit) with higher risk of failure. Critically, fatigued participants chose the low-effort strategy more often than well-rested participants. As these studies were the first to operationalize the assumed cost-benefit analysis explicitly as a decision process, they hold important theoretical value for the field of cognitive fatigue. Nevertheless, decision-making methodologies as used by Holding have seldom been adopted in later fatigue research.

## Effort-based decision making: an emerging field

The separate field of decision neuroscience, which is particularly involved in studying decision processes, has recently shown a surge in interest in effort-based decisions. Inspired by animal studies on motivation (Salamone et al., 1991; Walton et al., 2002; Rudebeck et al., 2006), and economic theory on expected utility (Von Neumann and Morgenstern, 1944), researchers have started to investigate how humans integrate effort and reward information in their decisions to act (Botvinick et al., 2009; Treadway et al., 2009; Kurniawan et al., 2010; Prévost et al., 2010). Similar to fatigue theory, it is proposed that the choice to engage in an action results from a weighing of action-costs (e.g., effort) against the value of its outcomes (Westbrook and Braver, 2015; Kool et al., 2017; Shenhav et al., 2017). If the required effort is high, the decision maker may assign less value to a reward compared to when effort is low. In other words, reward value is discounted based on effort costs (Westbrook et al., 2013).

**Figure 1:**
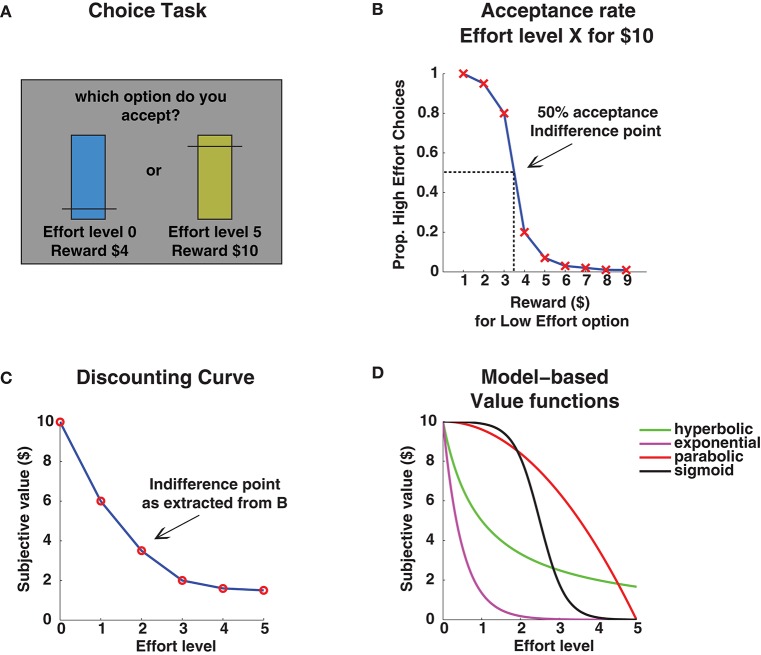
**(A)** Example choice trial, **(B)** Determination of indifference point, **(C)** Indifference points for different effort levels, **(D)** Theoretical discount functions.

A variety of paradigms has been developed to assess the influence of effort and reward on decision making (for reviews see Chong et al., 2016; Pessiglione et al., 2017). Typically, participants are given choices between performance of an effortful task, in return for a large reward, or a non-effortful task for a lower reward (Figure 1A). By sampling an individual's preference over a wide range of reward levels, a slope can be calculated that plots the willingness to accept the effort (Figure 1B). An indifference point, i.e., the reward level at which the effortful and non-effortful rewards are deemed equally attractive, can be determined over a range of effort levels, forming a discounting curve (Figure 1C). Much like the decision-making paradigms used by Holding and colleagues (Shingledecker and Holding, 1974; Holding et al., 1983), effort-discounting relies on the individual's choice of action. The particular advantage lies in the potential to estimate an integrated effort-reward value and its changes under conditions such as fatigue, as proposed by Kanfer (2011).

A major methodological asset is that, through computational modeling, normative mathematical functions can be fit to behavioral choices (Figure 1D; Prévost et al., 2010; Klein-Flügge et al., 2015; Zénon et al., 2016; Chong et al., 2017). This helps to formalize predictions and extrapolate beyond the specific test set. Moreover, it allows to incorporate biologically plausible cost-functions, which greatly improves predictions of behavioral and neuroimaging/physiological data (Manohar et al., 2015; Klein-Flügge et al., 2016; Le Bouc et al., 2016). It is still debated whether the effort-costs can be captured by a singular value-function (particularly in the domain of cognitive effort; Białaszek et al., 2017; Chong et al., 2017; Massar et al., 2018), however, computational approaches can strongly aid to generate testable hypotheses about the distinct cognitive and neurobiological mechanisms affected (e.g., motivation versus capacity deficits; Le Bouc et al., 2016).

## Fatigue and effort-based decision making: a way forward

We propose that fatigue research could greatly benefit from more integration of methods from decision neuroscience. Particularly, predictions from fatigue models, that have thus far remained untested could be directly examined. A starting point would be to model the effort-value function before and after a fatigue induction. A central prediction from fatigue theories would be that, under fatigue, the integrated effort-value function would be shifted toward a diminished preference for effort (Kanfer, 2011). Similar findings have been reported in related areas like sleep deprivation and physical fatigue (Libedinsky et al., 2013; Iodice et al., 2017; Massar et al., 2018), but not yet for cognitive fatigue.

Importantly, it could be tested how changes in effort-discounting relate to alterations in task performance and changes in the subjective sensation of fatigue. Several models describe subjective fatigue (or associated discomfort and effort sensation) as an internal signal that biases behavior away from non-rewarding activities (Boksem and Tops, 2008; van der Linden, 2011; Kurzban et al., 2013). Models differ slightly in the exact role they propose that subjective fatigue has in the effort-reward weighing process, but all would predict that higher felt fatigue would relate to stronger effort-avoidance. It should be noted that a recent study that looked at this relationship, did not find significant correlations between effort-discounting and subjective fatigue (Benoit et al., in review). Despite this initial negative result, we argue that more research is needed to further test the above described possibilities.

With regard to performance, effort-discounting information could be used further delineate the effects of time-on-tasks versus recovery. Studies on physical effort have already modeled how fatigue accumulates with prolonged muscle contraction, and dissipates with rest (Meyniel et al., 2013), and how this changes over different effort and reward conditions. Similarly, for cognitive performance, decline with time-on-task, and recovery with rest have been topics of investigation (Ross et al., 2014; Lim and Kwok, 2016), but have not yet been modeled in light of effort-reward tradeoffs. A similar modeling approach could be used to describe fluctuations in cognitive performance over time, formalizing the effort management process as proposed by Hockey (1997, 2011).

Furthermore, an important area where effort-based decision methods could inform fatigue research is in examining the neural mechanisms underlying motivation decline. Neuro-economic studies have revealed a particular set of brain areas and networks involved in reward valuation, effort evaluation, and subjective value computation (e.g., ventral striatum, anterior insula, anterior cingulate cortex: Prévost et al., 2010; Bartra et al., 2013; Meyniel et al., 2013; Apps et al., 2015; Massar et al., 2015; Klein-Flügge et al., 2016), many of which converge with the neural framework of fatigue as proposed by Boksem and Tops (2008). Any shifts in behavioral preference during fatigue, would likely be accompanied by alterations in the way these neural systems would interact. Studying how such changes in neural function would relate to behavioral preference and performance decrement may provide key insights into the motivational effects of fatigue.

A related question is whether effects of fatigue would transfer across tasks, or alternatively be more task-specific. Different tasks have been used in effort-based decision studies (e.g., working memory, task-switching, sustained attention; Kool et al., 2010; Westbrook et al., 2013; Apps et al., 2015; Massar et al., 2016), and different tasks have resulted in distinct carry-over effects after fatigue induction (Massar et al., 2010). It is therefore possible that any changes in effort-preference would depend on the overlap in brain circuitry that is being taxed during fatigue induction (Blain et al., 2016).

## Conclusion

In this paper, we have outlined how motivation and effort considerations have long been influential in theoretical models of fatigue, and how these ideas hold strong parallels with more recent theories of effort-based decision making. Although some researchers have started to explore the overlap, both fields still largely exist as separate areas. We would urge for a much stronger integration of these fields, and the adoption of decision methods to inform fatigue research. We are not the first to advocate the theoretical link between these fields, but we argue that the methodological development of effort-based decision making has now advanced to such extent that it can strongly accelerate insights in fatigue research.

## Author contributions

SM drafted the first version of the manuscript. DvdL and ÁC participated in writing and critical revision of the manuscript. All authors approved the final version.

### Conflict of interest statement

The authors declare that the research was conducted in the absence of any commercial or financial relationships that could be construed as a potential conflict of interest. The reviewer JJ and handling editor declared their shared affiliation.
